# Analysis of influenza surveillance results in Sichuan Province in China, 2023–2025

**DOI:** 10.3389/fmed.2026.1820896

**Published:** 2026-05-05

**Authors:** Lijun Zhou, Zhirui Li, Xingyu Zhou, Renxing Xie, Jiushun Zhou, Shuang Dong, Biao Yang, Fengmiao Hu, Xiuwei Cheng, Yao Ma, Feifei Yao, Chongkun Xiao

**Affiliations:** 1 Sichuan Center for Disease Control and Prevention, Chengdu, Sichuan, China; 2 Sichuan Tianfu New Area Public Health Center, Chengdu, China

**Keywords:** disease surveillance, influenza, influenza virus, influenza-like illness, sentinel surveillance

## Abstract

**Objectives:**

An analysis of influenza surveillance data from sentinel hospitals throughout five areas of Sichuan Province from 2023 to 2025 will clarify influenza epidemic trends, therefore providing significant support for preventative and control methods.

**Method:**

The Influenza monitoring Information System of China gathered Influenza-Like Illness (ILI) data from sentinel hospitals for respiratory monitoring in Sichuan Province. Descriptive epidemiological techniques and cross-correlation analysis were used to evaluate lag displacement across areas in the Chengdu Plain. Dynamic Time Warping (DTW) methods evaluated the morphological variance of epidemic curves, whereas spatio-temporal coupling coefficients quantified the strength of collaborative diffusion across locations.

**Results:**

During a three-year period, the province’s influenza positivity rate averaged 16.70%, reaching a maximum of 19.31% in 2023, with slight variations in the following 2 years (*p* < 0.001). The predominant circulating strains demonstrated a temporal shift from A(H3N2) in 2023 to A(H1N1)pdm09 in 2024, before returning to A(H3N2) in 2025, with the B(Victoria) lineage co-circulating throughout the study period. The population mostly consisted of children aged 0–14, with the 0–4 age group being the biggest proportion, fluctuating between 40 and 60% overall. A spatial analysis revealed that 56.3% of provincial cases originated from the Chengdu Plain, with influenza spreading from central to peripheral areas.

**Conclusion:**

The spread of influenza in Sichuan Province is shaped by natural terrain and human mobility, resulting in a pattern where outbreaks propagate from the Chengdu Plain to surrounding areas. Future preventative and control measures should effectively use the 1 week epidemiological lag time across regions to establish a geographically diversified, sub-regional collaborative early warning system. It is essential to prioritize the enhancement of pathogen genomes and the monitoring of extensive data in areas with high cross-regional population mobility, hence enabling precise interventions and resource allocation within the province.

## Introduction

Influenza is an acute respiratory infection that is caused by influenza viruses, which are present on a global scale year-round (1). It is characterized by an abrupt onset, rapid transmission, a wide-ranging impact, and elevated incidence rates (2). In densely populated regions, influenza viruses are primarily transmitted through droplet and contact transmission, which allows for rapid and extensive transmission (3). In the past, influenza pandemics have occurred, posing a persistent and substantial hazard to public health and human health (4). According to the most recent estimates from the World Health Organization, influenza cases worldwide occur annually at a rate of approximately 1 billion (5). Globally, seasonal influenza results in 3–5 million severe cases and 290,000 to 650,000 fatalities from influenza-related respiratory infections each year (6). The persistently elevated incidence poses a substantial challenge to global health systems and jeopardizes public health. Preventative and control measures are complicated by the unpredictable character of influenza viruses (7, 8). The influenza virus’s genome is composed of eight gene segments, and their rearrangement is a critical mechanism for the rapid generation of new viral strains (9). Phenotypic changes can result from genetic exchange due to co-infection by viruses, which can modify viral antigenicity and enable the virus to evade detection by the human immune system (10). The utilization of both vaccines and medications is complicated by this viral heterogeneity (11). In 2019, non-pharmaceutical interventions (NPIs) such as mask utilization, social separation, and restrictions on gatherings were rigorously enforced in response to the COVID-19 pandemic (12). In addition to effectively mitigating the transmission of SARS-CoV-2, these methods significantly altered the global ecology and seasonal transmission dynamics of respiratory viruses, resulting in a significant decrease in influenza activity in numerous regions throughout 2020–2021 (13). Influenza activity has experienced a resurgence to varying degrees following the progressive relaxation of non-pharmaceutical interventions following the COVID-19 pandemic in early 2023 (14).

Although influenza has been integrated into global infectious disease management frameworks, with the World Health Organization (WHO) leading the establishment of the Global Influenza Surveillance and Response System (GISRS) and countries progressively improving their own surveillance systems, it continues to impose a substantial disease burden and demand a substantial amount of healthcare resources (7, 15). Its effects are most pronounced in high-risk populations, including the elderly, infants, and those with underlying medical conditions (16). The essential foundation for early outbreak detection, trend assessment, and strategy formulation is influenza surveillance (7). The proportion of ILI and the influenza positive rate among ILI Specimens (ILI-PR) are critical monitoring indicators that reflect the capabilities of laboratory surveillance and the intensity of transmission, respectively. These metrics, when combined, offer a precise representation of the actual circulation of the influenza virus (17).

**Figure 1:**
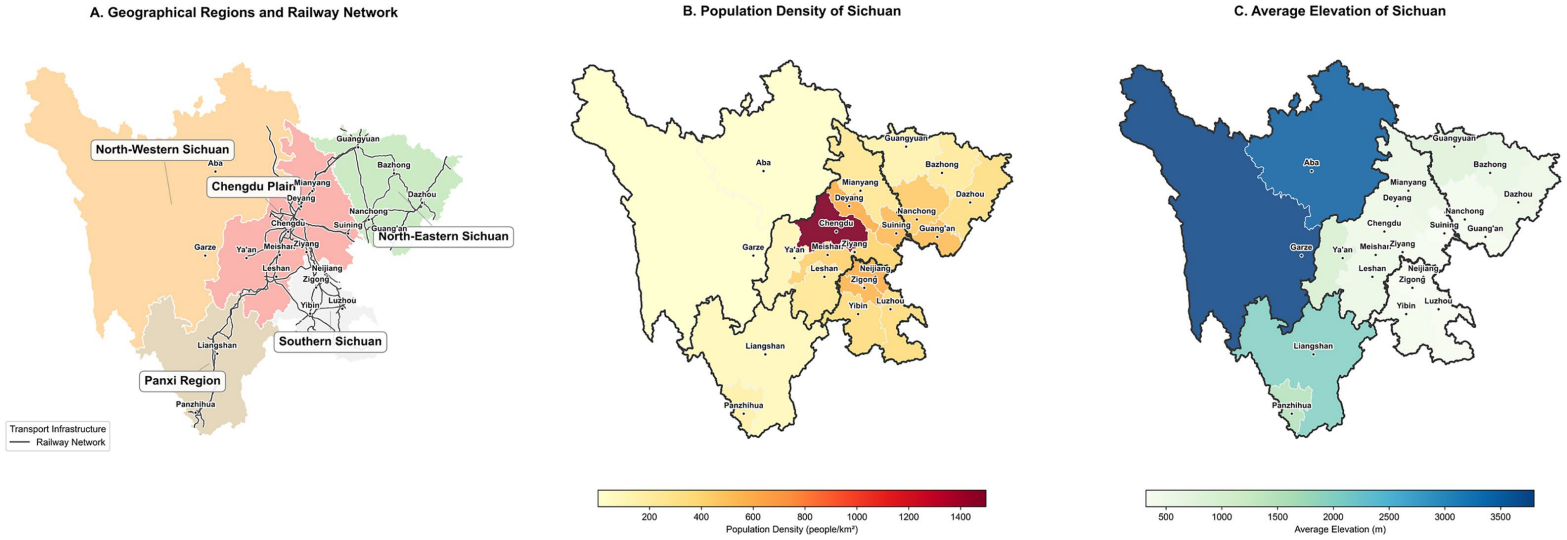
Overview of the study area. **(A)** Geographical regions and railway network, **(B)** population density, and **(C)** average elevation of Sichuan Province.

China has established a nationwide influenza surveillance network to collect comprehensive and continuous data on viral mutations and epidemic trends in real time (18). Sichuan Province has implemented integrated surveillance of respiratory pathogens in all 21 prefecture-level communities and autonomous prefectures since 2009, resulting in comprehensive coverage of sentinel hospitals. The province of Sichuan, which spans the Qinghai-Tibet Plateau and Sichuan Basin, is characterized by a diverse topography, climate, and population distribution, which may have an impact on influenza epidemiology (19). Consequently, the province is divided into five regions: Chengdu Plain, Southern Sichuan, North-eastern Sichuan, Panxi region, and North-western Sichuan, in accordance with the provisions of the Territorial Space Planning (2021–2035) issued by the Department of Natural Resources (Figure 1) (20). The eastern basin region, which includes the Chengdu Plain, North-Eastern Sichuan, and South-Eastern Sichuan, featuring major cities like Chengdu, Mianyang, Nanchong, and Yibin, is densely populated and possesses a highly developed transport network of roads and railways. The frequent population movement within the regional economic sphere has facilitated the virus’s spread. Conversely, the western plateau and Panxi regions, exemplified by North-Western Sichuan (including Aba and Ganzi), exhibit low population density, with population mobility hindered by geographical obstacles and considerable distances; this geographical seclusion has objectively impeded the swift dissemination and proliferation of the virus.

Throughout the COVID-19 pandemic, many non-pharmaceutical interventions (NPIs)—including mask usage, social distance, travel limitations, and school closures—were extensively enacted (21). Despite these methods substantially impeding the overall transmission of seasonal influenza, a huge study gap persists in comprehending the intricate spatiotemporal dynamics of influenza in areas characterized by considerable geographical and demographic diversity, such as Sichuan Province. In the post-COVID period, the epidemiological landscape of respiratory diseases has significantly altered, with influenza reestablishing its dominance in China. Recent studies indicate a significant comeback of influenza A and B in Sichuan Province during the 2023–2024 influenza season, characterized by an increasing overall incidence trend and a notable epidemic peak in 2023 (22, 23). The changing spatiotemporal dynamics demonstrate that the influenza transmission network in this area is significantly influenced by geographical and demographic variables. In light of these post-pandemic changes in trends, there is an imperative requirement for ongoing and updated monitoring. This study seeks to clarify the recent evolution patterns of influenza epidemics and evaluate the lag-shift phenomenon across various regions of the Chengdu Plain. It will conduct a systematic analysis of influenza surveillance data from sentinel hospitals in five regions of Sichuan Province between 2023 and 2025, thereby offering significant scientific evidence to enhance geographically diversified sub-regional collaborative strategies for epidemic prevention and control. This would establish a scientific basis for developing early warning methods for seasonal influenza in Sichuan Province, while also enhancing essential technical skills and data support for addressing any future influenza pandemics.

## Method

### Data source

This study combined influenza surveillance data from five geographical regions in Sichuan Province (Chengdu Plain, Southern Sichuan, Northeastern Sichuan, Panxi, and Northwestern Sichuan) from January 1, 2023, to December 31, 2025. Data is sourced from the “China Influenza Surveillance Information System” gathered by healthcare professionals in accordance with the “National Influenza Surveillance Technical Guidelines” and the “National Acute Respiratory Infectious Disease Sentinel Surveillance Technical Protocol” (24). The following data components are essential: identification, gender, age, employment, domicile, onset date, specimen type, collecting hospital, collection date, reception date, receiving unit, testing date, and test result.

As per the testing procedure, respiratory specimens (pharyngeal samples) from cases of influenza-like illness collected at sentinel hospitals must be sent to the influenza surveillance network laboratory for influenza virus nucleic acid testing within 7 days of collection. Specimens that are to be delivered to the network laboratory within 2 days must be stored at a temperature between 2 and 8 °C. In the event that samples are unable to be transported to the laboratory within 2 days, they must be stored at a temperature of −70 °C or lower to ensure that they are sent to the appropriate network laboratory within 1 week. The influenza surveillance network laboratory must ascertain the influenza virus subtype or lineage within three working days of receiving samples from sentinel hospitals by employing nucleic acid testing procedures (Real-time PCR). (1) The proportion of Influenza-like Illness (ILI) is calculated by dividing the total number of outpatient and A&E visits during a given period by the total number of people attending outpatient and A&E departments. (2) Influenza Positive Rate among ILI Specimens (ILI-PR): the portion of the total number of ILI specimens tested in the laboratory that tested positive for the influenza virus.

### Outcome

This study examined two categories of outcome indicators: (1) influenza-like illness (ILI) visits reported by sentinel hospitals from their monitoring clinics, which include internal medicine outpatient/emergency departments, pediatric outpatient/emergency departments, and fever clinics, on a weekly basis; and (2) influenza virus nucleic acid test results from ILI specimens collected by sentinel hospitals and processed in network laboratories. Influenza-like illness (ILI) is defined by a fever (body temperature ≥ 38 °C) in conjunction with either a cough or hoarse throat.

To maintain the original temporal resolution and prevent artificial autocorrelation, all analyses were conducted exclusively on unprocessed weekly surveillance data. This study utilized cross-correlation analysis to assess the leading or lagging relationships across places to ascertain the pathways of influenza transmission in Sichuan Province. The epidemic curves for the other regions are calibrated along the time axis by reference the Chengdu Plain, which functions as the core of the province’s transportation network and population movement. The ideal lag time is determined by an iterative search within a cautious 8-week frame, selecting the time shift that produces the largest statistically significant positive cross-correlation coefficient (*ρ*) with *p* < 0.05. The lack of a clear lag-based transmission connection is suggested if none of the correlation coefficients inside the window are significant. We employed Dynamic Time Warping (DTW) to ascertain the smallest cumulative variance in epidemic development between two locations by nonlinearly aligning peak and trough values across sequences (25).

To evaluate the robustness of regional spatiotemporal linkages, the Spatio-temporal Coupling Coefficient (STCC) was calculated within the established framework of the entropy weight method (EWM)–based comprehensive evaluation model (26, 27):

$$\mathrm{STC}C_{i}=\omega_{1}\cdot \rho_{i}+\omega_{2}\cdot (1-\frac{\mathrm{La}g_{i}}{\mathrm{La}g_{\max}})+\omega_{3}\cdot (1-\frac{\mathrm{DT}W_{i}}{\max(\mathrm{DTW})})$$

All individual metrics ( ρ , Lag, and DTW) were standardized using min-max normalization to ensure comparability across different scales. Where $\rho_{i}$ denotes the greatest cross-correlation coefficient, $\mathrm{La}g_{i}$ represents the ideal lag time, and $\mathrm{DT}W_{i}$ signifies the DTW distance. The weighting method ( ω ) was objectively established by the Entropy Weight Method (EWM), which allocates weights according to the information entropy of each indicator across areas to reduce subjective bias. The STCC number ranges from 0 to 1, with a value nearing 1 indicating improved synchronization and more consistency in the propagation process. Additionally, to address sample variability, 95% confidence intervals (95% CI) for all STCC values were calculated using a non-parametric bootstrapping method with 1,000 iterations (28).

### Statistical analysis

The primary organization of monitoring data was accomplished using WPS Office software. The quantities of ILI specimens were compiled based on the date of sampling, while ILI case visits were recorded according to the time of presentation. The Pandas library, SciPy, and implementations of the DTW-python package were used to execute data cleansing and feature extraction with Python 3.9.23. At a significance threshold of α = 0.05, all tests were conducted.

## Results

### Epidemiological overview

The fundamental epidemiological summary for the years 2023 to 2025 is presented in Table 1. Between 2023 and 2025, national sentinel hospitals in Sichuan Province documented a total of 1,860,808 cases of influenza-like illness (ILI). A total of 126,246 ILI specimens were collected and tested, culminating in 21,077 influenza-positive results. The overall affirmative detection rate was 16.70%. The peak specimen positive rate was recorded in 2023 at 19.31% (6,935/35,905), which decreased to 15.57% (6,204/39,854) in 2024 and marginally increased to 15.72% (7,938/50,487) in 2025, according to annual trends. Chi-square analysis indicated statistically significant variations in positive detection rates across the years ( $X^{2}$ = 247.97, *p* < 0.0001).

**Table 1:** Basic epidemiological characteristics of influenza-like illness cases in Sichuan Province, 2023–2025.

Items	2023	2024	2025	Total	Statistical analysis
Total number of ILI cases	671,121	594,760	594,927	1,860,808	
Number of specimens collected	35,905	39,854	50,487	126,246	
Number of positive detections	6,935	6,204	7,938	21,077	
Overall positive rate (%)	19.31%	15.57%	15.72%	16.70%	χ^2^ = 247.97, *p* < 0.0001

**Figure 2:**
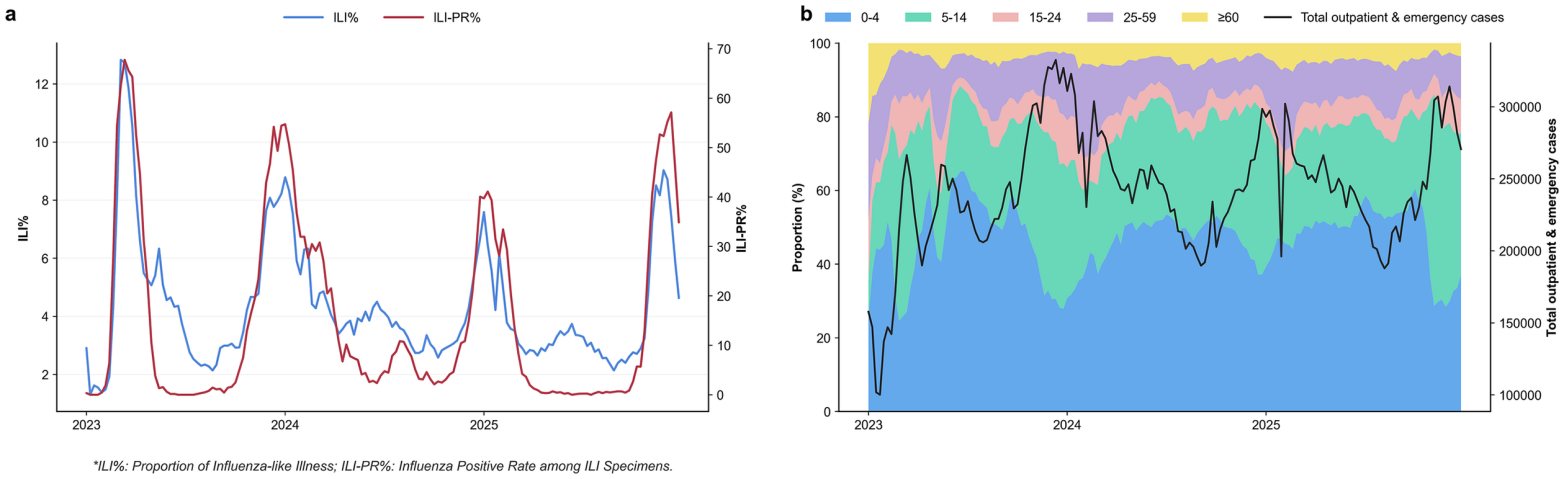
**(a)** Dynamic trend chart of influenza-like illness proportion (ILI%) and nucleic acid test positivity rate (ILI-PR), 2023–2025. **(b)** Changes in age composition and visit volume of outpatient and emergency cases in influenza surveillance clinics, 2023–2025.

The comprehensive influenza surveillance findings for 2023–2025 are depicted in Figure 2a, which suggests a strong correlation in fluctuation patterns between the ILI% percentage and the nucleic acid positive rate. The epidemic intensity peaked in 2023, with an ILI% of approximately 13% and a positivity rate of approximately 70%, the highest levels recorded during the monitoring period. The anticipated zenith ranges for 2024 and 2025 have been reduced to 7–9% and 40–60%, respectively. The nucleic acid positive rate exhibited a significantly greater degree of variability than the ILI% during the monitoring period. The Eastern Basin experienced a high level of epidemic intensity as a result of its dense population and high mobility. Conversely, the Western Plateau and Panxi regions manifested a lower level of epidemic intensity as a result of their sparse population density and natural obstacles. The age distribution of outpatient and emergency patients in monitored clinics from 2023 to 2025 is depicted in Figure 2b. The 0–4 age cohort accounted for the largest proportion, with the 0–14 age demographic being the most prevalent, with a total range of 40 to 60%. Subsequently, 20–40% of the population was between the ages of 5 and 14. The age demographic of 25–59 comprised approximately 10–20% of the population; however, the 15–24 and ≥60 age categories held the lowest percentages. The simultaneous ILI percentage and nucleic acid positive rate are strongly correlated with the trend in total outpatient and emergency visits.

### Pathogenic detection overview

**Figure 3:**
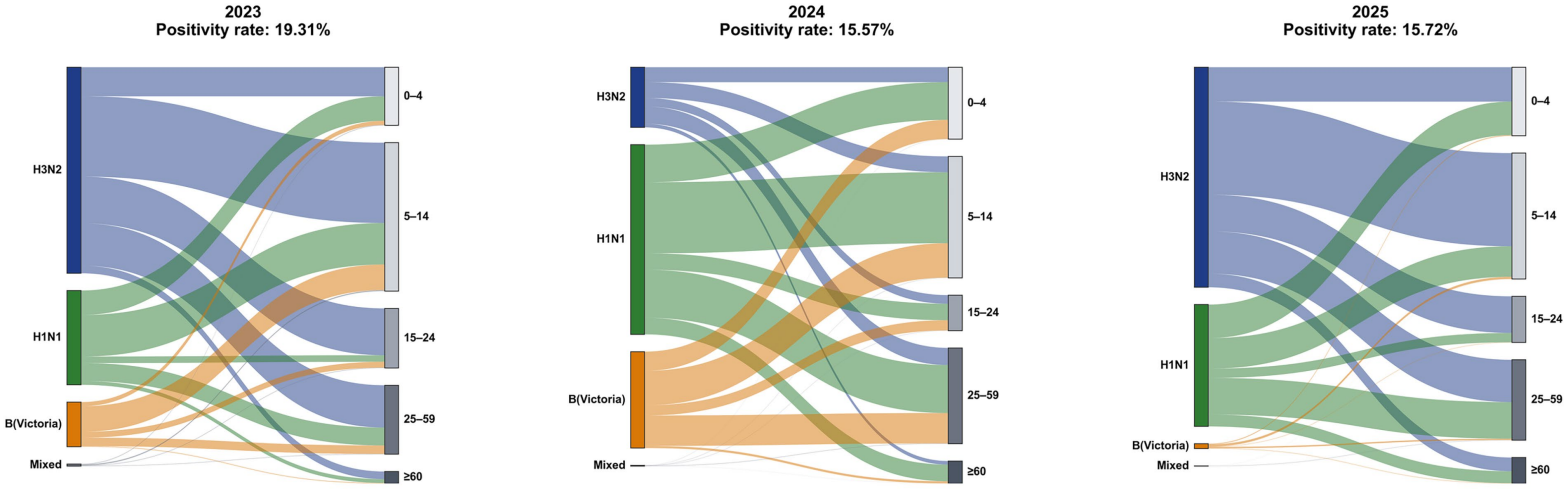
Distribution of nucleic acid test results for ILI specimens in Sichuan Province, 2023–2025.

The provincial pathogen detection summary for the years 2023 to 2025 is depicted in Figure 3. A cumulative total of 126,246 specimens for influenza-like symptoms were tested across the province over the course of 3 years, resulting in 21,077 nucleic acid-positive findings. The cumulative positivity rate was 16.70%. The annual positivity rates for 2023 were 19.31, 15.57% for 2024, and 15.72% for 2025. The predominant influenza virus strains exhibited a cyclical pattern of A(H3N2)—A(H1N1)—A(H3N2) throughout the surveillance period. 2023: A(H3N2) was prevalent at 59.38%; 2024: A(H1N1) became dominant at 54.69%; 2025: A(H3N2) regained supremacy at 63.43%. In terms of age distribution, cases across all years and subtypes were mostly concentrated among school-aged children (5–14 years) and young adults (25–59 years), whereas the ≥60 age group consistently represented a minimal proportion across all years and subtypes.

Supplementary Table S1 illustrates that there is a significant regional disparity in the distribution of cases that resemble influenza. The Chengdu Plain is home to the province’s highest concentration of ILI cases, with a cumulative total of 1,047,800 cases reported over a three-year period, which accounts for 56.31% of the statewide total. In 2025, the specimen positive rate suffered a decline from 20.53% in 2023 to 16.71%. The North-eastern and South Sichuan regions closely followed. The province’s surveillance intensity has increased annually, with specimen collection increasing from 35,900 samples in 2023 to 50,500 samples in 2025—a 40.67% increase. The aggregate positive rate remained within the 15–20% range, despite the substantial increase in sampling volume. From 2023 to 2025, there were 126,246 positive nucleic acid test findings for the influenza virus, including 63,942 men and 62,304 females. The predominant age group was 0–4 years (42,356 cases), followed by 5–14 years (32,646 cases) and 25–59 years (26,220 cases). The primary specimen type was throat swabs, totaling 126,244 samples, whereas just two nasal swabs were collected. The occupational distribution was mostly comprised of homemakers and jobless individuals (7,083 instances), succeeded by retired persons (5,852 cases) and those with unspecified jobs (7,840 cases). The virus subtypes demonstrated a clear pattern of yearly alternation: A(H3N2) predominated in 2023 and 2025, while A(H1N1) was prominent in 2024; the B(Victoria) subtype co-circulated to variable extents in all years.

### Spatio-temporal correlation results

The spatiotemporal correlation analysis results between the four sub-regions of Sichuan Province and the Chengdu Plain are presented in Table 2, which is based on raw weekly surveillance data. The cross-correlation analysis demonstrates a distinct centrifugal dissemination pattern that is centered on the Chengdu Plain. The Chengdu Plain was the most closely synchronized with Southern Sichuan and North-Eastern Sichuan during the 2023–2025 period. Both regions exhibited robust cross-correlation coefficients (ρ = 0.954 and 0.945, respectively). The most intensive coupling relationship with the hub was indicated by the spatiotemporal coupling coefficients (STCC) for these two regions, which reached 0.86 (95% CI: 0.84–0.89). North-Eastern Sichuan exhibited the maximum waveform similarity, as evidenced by the narrowest DTW distance (396.39). Conversely, the coupling intensity exhibits a gradual decrease as the complexity of the terrain and the distance between the locations increase. With an STCC of 0.78 (95% CI: 0.66–0.85), the Panxi Region demonstrated a stochastic latency to +1 week. In the same vein, North-Western Sichuan exhibited the highest DTW distance (549.38) and a latency to +1 week, resulting in an STCC of 0.82 (95% CI: 0.75–0.85). After accounting for temporal shifts, all regional cross-correlation coefficients exceeded 0.85, thereby verifying a high level of linear synchronization throughout the province. The inclusion of 95% confidence intervals derived from 1,000 bootstrap iterations further substantiates the statistical significance of the gradient in STCC values, which reflects the varying impact of demographic mobility and environmental forcing on influenza transmission dynamics relative to the Chengdu Plain.

**Table 2:** Spatio-temporal correlation and coupling analysis of influenza activity between Chengdu Plain and other regions in Sichuan Province (2023–2025).

Region (vs. Chengdu Plain)	Lag time (weeks)	Cross-correlation coefficient (ρ)	DTW distance	Spatio-temporal coupling coefficient
Southern Sichuan	+0	0.954	417.52	0.86 (0.84–0.89)
North-Eastern Sichuan	+0	0.945	396.39	0.86 (0.84–0.89)
Panxi Region	+1	0.889	444.38	0.78 (0.66–0.85)
North-Western Sichuan	+1	0.868	549.38	0.82 (0.75–0.85)

## Discussion

Influenza, as an acute respiratory infectious illness, has consistently been scrutinized for its epidemic characteristics (14). Non-pharmaceutical interventions decreased influenza activity levels in Sichuan Province during the COVID-19 pandemic. Following the removal of these restrictions, influenza activity progressively reverted to pre-pandemic levels. During influenza peaks, the aggregate of outpatient and emergency department visits demonstrated a closely aligned increase with the incidence of ILI cases. The time of their peaks was notably synchronized, and the length of peak activity was predominantly uniform. This trend validates the direct influence of influenza epidemics on outpatient and emergency medical care requests, aligning with results from many domestic and international influenza sentinel monitoring studies (29). During the three-year span, influenza positive rates shown considerable variability, reaching a zenith of 19.31% in 2023, thereafter stabilizing in 2024 and 2025. The disparities between the years were statistically significant (*p* < 0.001). This fluctuation in epidemic severity indicates the focused release and compensating process of post-pandemic population immune deficit. Following extended durations of little circulation, pre-existing immunity to seasonal influenza significantly diminished, resulting in the unprecedented epidemic peak recorded in 2023 (30). Regarding pathogen composition, the dominating strains throughout the monitoring period exhibited a distinct succession cycle aligned with the yearly distribution: A(H3N2) in 2023, A(H1N1) in 2024, and A(H3N2) once more in 2025. This succession pattern exemplifies the survival strategy of influenza viruses, shaped by immunological pressure from the population and antigenic drift. The A(H3N2) strain exhibited increased pandemic resistance, remaining prevalent in 2023 and re-emerging as the dominant virus in 2025. This is likely due to the heightened mutation rate of the A(H3N2) hemagglutinin (HA) gene among all influenza subtypes, facilitating the rapid replacement of current variants with new ones, hence enhancing its immune evasion capabilities relative to other subtypes. Alongside these influenza A subtypes, influenza B (Victoria) also co-circulated during the monitoring period, aligning with the data presented in the Results section (31).

The predominant cases of ILI in outpatient and emergency departments are children under 15 years, with the 0–4 age group representing the largest percentage (40–60%), whereas the ≥60 age group continuously exhibits a low incidence. Children have underdeveloped immune systems that lack specialized antibodies for new or mutated strains, rendering them considerably more vulnerable to influenza viruses compared to adults (32). From a sociological standpoint, this group is concentrated in highly crowded environments such as educational institutions and childcare centers, where proximity facilitates optimal conditions for viral transmission (33). Moreover, children frequently have more characteristic symptoms at the outset of illness, resulting in heightened parental urgency for medical attention, hence augmenting the percentage of pediatric ILI cases in outpatient and emergency environments (34). The strong association between total outpatient/emergency visits and ILI cases suggests that kid epidemics function as both early markers of community transmission and significant contributors to seasonal pressure on the healthcare system. The elevated prevalence of adult cases (ages 25–59) may be attributed to their status as the primary working age demographic, resulting in extensive interpersonal interactions that objectively heighten their risk of infection and transmission (35). The consistently low percentage of individuals over 60 in ILI monitoring does not accurately indicate a low infection incidence; instead, the constraints of passive surveillance techniques and many variables obscure the actual illness burden (36). Older persons in China typically have a strong tolerance for minor symptoms and frequently choose to procure medicine independently or seek treatment at local primary care institutions instead of attending major sentinel hospitals (37). Secondly, post-infection, older adults frequently exhibit atypical influenza-like illness symptoms, such as diminished fever, instead displaying lethargy or worsening of pre-existing chronic conditions; consequently, they are often misdirected to other specialist departments during triage, resulting in under-reporting in surveillance (38). Moreover, the health status of elderly patients can deteriorate swiftly, often culminating in severe pneumonia and necessitating direct admission through the emergency department or to the ICU, thereby entirely circumventing the ILI monitoring points in general outpatient and emergency departments (39). Therefore, while analyzing passive surveillance data from outpatient and emergency departments, it is crucial to acknowledge the substantial underestimating of the actual impact of influenza on the senior demographic.

This work quantitatively demonstrates, by cross-correlation and DTW algorithm analysis, that influenza transmission in Sichuan Province displays a core-synchronous, edge-mildly-lagged pattern focused on the Chengdu Plain. The Sichuan Basin, encircled by mountains, possesses climatic conditions conducive to the prolonged airborne survival of influenza viruses; concurrently, the Chengdu Plain, as the transportation hub and population center of the province, acts as the principal epicenter for influenza epidemics. Southern Sichuan and North-Eastern Sichuan exhibited near-perfect spatiotemporal synchronization with the Chengdu Plain, characterized by an ideal lag time of 0 weeks, elevated cross-correlation coefficients (ρ > 0.94), and robust Spatio-temporal Coupling Coefficients (STCC = 0.86). This suggests that influenza activity in these two basin regions is almost contemporaneously linked with the Chengdu Plain, exhibiting no substantial temporal lag. The synchronized patterns between these regions and the Chengdu Plain demonstrate the increased efficacy of viral transmission resulting from post-pandemic network integration in Sichuan. This phenomenon corresponds with spatial transmission patterns in infectious disease dynamics, where viruses generally originate in densely populated, economically robust core cities and subsequently disseminate to neighboring secondary economic zones through high-mobility transportation corridors like high-speed rail and long-distance busses (40). The intercity commute, high-speed rail systems, and substantial population movements across regions have markedly diminished obstacles to cross-regional viral transmission (41). In this situation, distance diminishes its efficacy in mitigating influenza transmission. The real-time monitoring data from the Chengdu Plain may serve as both an early warning indication for Southern Sichuan and North-Eastern Sichuan and as a nearly equal real-time epidemiological representation. Both core and neighboring basin regions should adopt a cohesive, coordinated approach in their pandemic response initiatives. Conversely, the Panxi Region and North-Western Sichuan demonstrated a minor but persistent delay of 0 to 1 week compared to the Chengdu Plain, along with marginally reduced cross-correlation coefficients and STCC values. The western plateau and Panxi region, characterized by high elevation, low population density, and natural mountain barriers, provide a geographical shield that limits rapid viral spread and penetration. The notable morphological heterogeneity and strong epidemiological independence seen in North-Western Sichuan suggest the potential existence of unique adaptive cycle patterns in this region. The identified geographic gradient effect—where the Chengdu Plain and adjacent basin regions exhibit complete synchronization, and western marginal areas display a 1 week delay—indicates that provincial influenza prevention and control efforts should prioritize enhanced active monitoring in the core Chengdu Plain and surrounding basin areas, while also implementing customized strategies to reduce imported cases in the western regions. Observing fluctuation patterns in the Chengdu Plain can provide a preparedness timeframe of 1 week for epidemic prevention in the western peripheral areas. This discovery provides a robust basis for creating a multi-tiered, regionally customized influenza early warning system across the province.

Despite the fact that this investigation has disclosed the spatio-temporal dynamics of influenza epidemics in Sichuan Province, there are still numerous constraints. Initially, the immense geographical expanse and complex topography of the north-western Sichuan and Panxi regions have resulted in a predominant concentration of extant national-level sentinel hospitals in central cities. This could lead to inadequate coverage of the monitoring network in vast, remote regions, which could introduce a certain degree of spatial sampling bias when evaluating the overall epidemic intensity in these regions. Consequently, it may be challenging to fully capture the hidden transmission chains of the virus at the grassroots community level. Secondly, this study has not yet conducted a comprehensive analysis of the adaptive differences among various influenza virus subtypes under varying temperature and humidity gradients, and it lacks high-resolution continuous meteorological data. Consequently, it is unable to fully elucidate the mechanisms by which natural environmental factors drive the rotation of specific strains. Additionally, our model did not include comprehensive indicators of population mobility or regional influenza vaccination coverage rates. The susceptibility of local populations is significantly impacted by the heterogeneity of vaccine-induced immune barriers. Additionally, the absence of high-precision mobility indicators has restricted our ability to conduct a comprehensive causal interpretation of the spatiotemporal coupling coefficient (STCC) between regions, which makes it challenging to accurately quantify the precise contribution of socio-economic interactions to epidemic synchronization. However, it must be acknowledged that the temporal lead observed in the outbreak on the Chengdu Plain does not entirely provide conclusive evidence of a causal direction of transmission. Although the identified centrifugal gradient is consistent with Chengdu’s status as a regional transport hub, this pattern could also be explained by common external drivers, such as an initial source event for influenza transmission or shared environmental influences. In this context, the temporal lag in regional outbreaks may stem from differences in population susceptibility or variations in local surveillance capabilities, rather than direct inter-regional transmission. Future research should incorporate high-resolution genomic data into geographical analyses or undertake detailed estimates of population mobility.

## Conclusion

This research methodically examined influenza surveillance data from Sichuan Province from 2023 to 2025. The severity of influenza reached its zenith in 2023 may as a result of the concentrated release of immunity debt, then stabilizing. The predominant strains switched cyclically between A(H3N2) and A(H1N1). The vulnerable demographic, especially children aged 0–14 years (notably the 0–4 age group), represented the principal strain on healthcare facilities and acted as the primary catalyst for influenza outbreaks throughout the province. The spatial dispersion of influenza has a pattern emanating from the Chengdu Plain. Areas with efficient mobility, like southern Sichuan, demonstrate exceptionally high transmission synchrony with the Chengdu Plain. Conversely, northeastern Sichuan and the Panxi area, sequestered by natural impediments, exhibit considerable epidemic delays and autonomy. The spatiotemporal progression of influenza in Sichuan Province is influenced by pathogen variety, social dynamics of the population, and natural geographical conditions. Future preventive and control initiatives must emphasize the establishment of a tiered collaborative early warning system informed by regional geographical diversity.

## Data Availability

Requests to access these datasets should be directed to Chongkun Xiao, fangy27001@163.com.
