# Supplementary material for: Analysis of influenza surveillance results in Sichuan Province in China, 2023–2025
Source: Front Med (Lausanne). 2026 May 5;13:1820896. doi: 10.3389/fmed.2026.1820896 (PMC13183819; doi:10.3389/fmed.2026.1820896)
Supplement: Supplementary file 1 [file Table_1.docx]

**Table S1a. Influenza-like illness (ILI) surveillance data by region, age group and year in Sichuan, 2023–2025**

| **Region** | **Age Group** | **ILI Cases** | | | **ILI Specimens Collected** | | | **ILI Positive Detections** | | | **ILI Positive Rate (%)** | | |
| --- | --- | --- | --- | --- | --- | --- | --- | --- | --- | --- | --- | --- | --- |
|  | **Year** | **2023** | **2024** | **2025** | **2023** | **2024** | **2025** | **2023** | **2024** | **2025** | **2023** | **2024** | **2025** |
| **Chengdu Plain** | 0–4 | 139,739 | 131,216 | 142,384 | 5,830 | 5,933 | 7,577 | 602 | 564 | 693 | 10.33 | 9.51 | 9.15 |
|  | 5–14 | 134,551 | 116,088 | 125,660 | 5,042 | 6,024 | 6,257 | 1,571 | 1,206 | 1,573 | 31.16 | 20.02 | 25.14 |
|  | 15–24 | 35,130 | 23,040 | 23,601 | 1,965 | 1,514 | 1,751 | 625 | 266 | 371 | 31.81 | 17.57 | 21.19 |
|  | 25–59 | 50,959 | 45,402 | 38,855 | 3,791 | 3,765 | 3,792 | 807 | 794 | 720 | 21.29 | 21.09 | 18.99 |
|  | ≥60 | 12,284 | 15,480 | 13,411 | 1,593 | 1,682 | 2,065 | 136 | 170 | 226 | 8.54 | 10.11 | 10.94 |
|  | **Subtotal** | **372,663** | **331,226** | **343,911** | **18,221** | **18,918** | **21,442** | **3,741** | **3,000** | **3,583** | **20.53** | **15.86** | **16.71** |
| **Southern Sichuan** | 0–4 | 43,186 | 28,213 | 30,086 | 2,017 | 2,373 | 3,751 | 143 | 143 | 343 | 7.09 | 8.01 | 9.14 |
|  | 5–14 | 37,599 | 19,626 | 21,144 | 1,854 | 2,324 | 2,348 | 565 | 377 | 614 | 30.47 | 16.22 | 26.27 |
|  | 15–24 | 14,185 | 8,845 | 8,481 | 740 | 815 | 1,203 | 291 | 206 | 292 | 39.32 | 25.28 | 24.27 |
|  | 25–59 | 15,480 | 13,743 | 12,485 | 842 | 1,452 | 1,730 | 201 | 454 | 431 | 23.87 | 31.27 | 24.91 |
|  | ≥60 | 5,004 | 4,928 | 4,686 | 245 | 329 | 786 | 20 | 77 | 107 | 8.16 | 23.4 | 13.61 |
|  | **Subtotal** | **115,454** | **73,555** | **76,882** | **5,698** | **7,293** | **9,818** | **1,220** | **1,304** | **1,787** | **21.41** | **17.88** | **18.2** |
| **North-eastern Sichuan** | 0–4 | 59,715 | 64,021 | 64,155 | 1,812 | 2,117 | 3,428 | 165 | 184 | 341 | 9.11 | 8.69 | 9.95 |
|  | 5–14 | 38,599 | 32,923 | 41,436 | 1,326 | 1,545 | 1,944 | 424 | 269 | 521 | 31.98 | 17.41 | 26.8 |
|  | 15–24 | 10,369 | 6,855 | 7,034 | 783 | 845 | 1,465 | 182 | 97 | 276 | 23.24 | 11.48 | 18.84 |
|  | 25–59 | 14,053 | 15,248 | 15,480 | 1,245 | 1,697 | 3,950 | 204 | 210 | 553 | 16.39 | 12.37 | 14 |
|  | ≥60 | 4,874 | 6,441 | 6,347 | 928 | 1,190 | 2,343 | 56 | 82 | 215 | 6.03 | 6.89 | 9.18 |
|  | **Subtotal** | **127,610** | **125,488** | **134,452** | **6,094** | **7,394** | **13,130** | **1,031** | **842** | **1,906** | **16.92** | **11.39** | **14.52** |
| **Panxi region** | 0–4 | 26,134 | 29,594 | 16,922 | 1,875 | 2,023 | 1,871 | 144 | 219 | 154 | 7.68 | 10.83 | 8.23 |
|  | 5–14 | 12,858 | 15,690 | 10,896 | 903 | 728 | 606 | 220 | 152 | 122 | 24.36 | 20.88 | 20.13 |
|  | 15–24 | 3,812 | 2,428 | 1,677 | 153 | 140 | 185 | 47 | 30 | 34 | 30.72 | 21.43 | 18.38 |
|  | 25–59 | 6,727 | 7,493 | 4,280 | 473 | 504 | 461 | 62 | 113 | 53 | 13.11 | 22.42 | 11.5 |
|  | ≥60 | 2,803 | 1,775 | 1,327 | 195 | 304 | 424 | 14 | 59 | 37 | 7.18 | 19.41 | 8.73 |
|  | **Subtotal** | **52,334** | **56,980** | **35,102** | **3,599** | **3,699** | **3,547** | **487** | **573** | **400** | **13.53** | **15.49** | **11.28** |
| **North-western Sichuan** | 0–4 | 865 | 1,426 | 1,233 | 728 | 511 | 510 | 108 | 128 | 37 | 14.84 | 25.05 | 7.25 |
|  | 5–14 | 915 | 1,299 | 941 | 692 | 641 | 412 | 187 | 167 | 52 | 27.02 | 26.05 | 12.62 |
|  | 15–24 | 486 | 587 | 539 | 205 | 372 | 428 | 45 | 35 | 83 | 21.95 | 9.41 | 19.39 |
|  | 25–59 | 657 | 1,559 | 1,172 | 552 | 918 | 1,048 | 104 | 142 | 83 | 18.84 | 15.47 | 7.92 |
|  | ≥60 | 137 | 840 | 695 | 116 | 108 | 152 | 12 | 13 | 7 | 10.34 | 12.04 | 4.61 |
|  | **Subtotal** | **3,060** | **5,711** | **4,580** | **2,293** | **2,550** | **2,550** | **456** | **485** | **262** | **19.89** | **19.02** | **10.27** |
| **Total** |  | **671,121** | **594,760** | **594,927** | **35,905** | **39,854** | **50,487** | **6,935** | **6,204** | **7,938** | **19.31** | **15.57** | **15.72** |

**Table S1b. Influenza virus nucleic acid positive detection in different years from 2023 to 2025**

| **Year** | **Month** | **A(H3N2)** | **A(H1N1)** | **B(Victoria)** | **Mixed** | **Total Positive** | **No. Tested** | **Positive Rate (%)** |
| --- | --- | --- | --- | --- | --- | --- | --- | --- |
| **2023** | January | 1(33.33) | 2(66.67) | 0(0) | 0(0) | 3 | 2839 | 0.11 |
|  | February | 258(48.22) | 276(51.59) | 0(0) | 1(0.19) | 535 | 3131 | 17.09 |
|  | March | 1295(57.05) | 957(42.16) | 2(0.09) | 16(0.70) | 2270 | 3508 | 64.71 |
|  | April | 612(53.17) | 526(45.70) | 1(0.09) | 12(1.04) | 1151 | 2930 | 39.28 |
|  | May | 66(54.10) | 55(45.08) | 1(0.82) | 0(0) | 122 | 3174 | 3.84 |
|  | June | 0(0) | 2(100.00) | 0(0) | 0(0) | 2 | 2550 | 0.08 |
|  | July | 1(50.00) | 1(50.00) | 0(0) | 0(0) | 2 | 2779 | 0.07 |
|  | August | 15(65.22) | 6(26.09) | 2(8.70) | 0(0) | 23 | 2827 | 0.81 |
|  | September | 22(68.75) | 2(6.25) | 8(25.00) | 0(0) | 32 | 2711 | 1.18 |
|  | October | 210(75.00) | 2(0.71) | 67(23.93) | 1(0.36) | 280 | 3146 | 8.9 |
|  | November | 602(71.92) | 21(2.51) | 213(25.45) | 1(0.12) | 837 | 3039 | 27.54 |
|  | December | 1036(61.74) | 34(2.03) | 596(35.52) | 12(0.72) | 1678 | 3271 | 51.3 |
|  | **Subtotal** | 4118(59.38) | 1884(27.17) | 890(12.83) | 43(0.62) | 6935 | 35905 | 19.31 |
| **2024** | January | 664(41.60) | 52(3.26) | 871(54.57) | 9(0.56) | 1596 | 3605 | 44.27 |
|  | February | 212(25.39) | 121(14.49) | 499(59.76) | 3(0.36) | 835 | 2798 | 29.84 |
|  | March | 113(14.97) | 350(46.36) | 290(38.41) | 2(0.26) | 755 | 2977 | 25.36 |
|  | April | 42(13.17) | 229(71.79) | 48(15.05) | 0(0) | 319 | 3134 | 10.18 |
|  | May | 6(3.73) | 149(92.55) | 6(3.73) | 0(0) | 161 | 2705 | 5.95 |
|  | June | 1(1.27) | 75(94.94) | 3(3.80) | 0(0) | 79 | 2691 | 2.94 |
|  | July | 5(2.04) | 240(97.96) | 0(0) | 0(0) | 245 | 3294 | 7.44 |
|  | August | 8(3.81) | 200(95.24) | 2(0.95) | 0(0) | 210 | 2582 | 8.13 |
|  | September | 6(5.94) | 93(92.08) | 2(1.98) | 0(0) | 101 | 3177 | 3.18 |
|  | October | 8(6.90) | 107(92.24) | 1(0.86) | 0(0) | 116 | 3814 | 3.04 |
|  | November | 7(2.07) | 331(97.93) | 0(0) | 0(0) | 338 | 4030 | 8.39 |
|  | December | 2(0.14) | 1446(99.79) | 1(0.07) | 0(0) | 1449 | 5047 | 28.71 |
|  | **Subtotal** | 1074(17.31) | 3393(54.69) | 1723(27.77) | 14(0.23) | 6204 | 39854 | 15.57 |
| **2025** | January | 2(0.13) | 1571(99.87) | 0(0) | 0(0) | 1573 | 4406 | 35.7 |
|  | February | 2(0.19) | 1026(99.52) | 2(0.19) | 1(0.10) | 1031 | 4238 | 24.33 |
|  | March | 3(1.59) | 168(88.89) | 18(9.52) | 0(0) | 189 | 4344 | 4.35 |
|  | April | 4(17.39) | 11(47.83) | 8(34.78) | 0(0) | 23 | 4074 | 0.56 |
|  | May | 11(68.75) | 0(0) | 5(31.25) | 0(0) | 16 | 3778 | 0.42 |
|  | June | 6(85.71) | 1(14.29) | 0(0) | 0(0) | 7 | 4141 | 0.17 |
|  | July | 4(40.00) | 6(60.00) | 0(0) | 0(0) | 10 | 4042 | 0.25 |
|  | August | 11(64.71) | 0(0) | 6(35.29) | 0(0) | 17 | 3524 | 0.48 |
|  | September | 27(62.79) | 3(6.98) | 13(30.23) | 0(0) | 43 | 4377 | 0.98 |
|  | October | 491(96.46) | 1(0.20) | 16(3.14) | 1(0.20) | 509 | 3811 | 13.36 |
|  | November | 2152(99.22) | 1(0.05) | 16(0.74) | 0(0) | 2169 | 4493 | 48.28 |
|  | December | 2322(98.77) | 0(0) | 25(1.06) | 4(0.17) | 2351 | 5259 | 44.7 |
|  | **Subtotal** | 5035(63.43) | 2788(35.12) | 109(1.37) | 6(0.08) | 7938 | 50487 | 15.72 |
|  | **Total** | 10227(48.52) | 8065(38.26) | 2722(12.91) | 63(0.30) | 21077 | 126246 | 16.7 |

**Table S1c. Influenza Virus Nucleic Acid Test Specimens by Year, Gender, Age Group and Specimen Type (2023–2025)**

| **Year** | **Male** | **Female** | **≥60** | **0–4** | **15–24** | **25–59** | **5–14** | **Nasal Swab** | **Throat Swab** |
| --- | --- | --- | --- | --- | --- | --- | --- | --- | --- |
| **2023** | 18378 | 17527 | 3077 | 12262 | 3846 | 6903 | 9817 | 0 | 35905 |
| **2024** | 20094 | 19760 | 3613 | 12957 | 3686 | 8336 | 11262 | 0 | 39854 |
| **2025** | 25470 | 25017 | 5770 | 17137 | 5032 | 10981 | 11567 | 2 | 50485 |
| **Total** | 63942 | 62304 | 12460 | 42356 | 12564 | 26220 | 32646 | 2 | 126244 |

**Table S1d. Influenza Virus Nucleic Acid Test Specimens by Occupation (2023–2025)**

| **Year** | **Nursery Workers & Babysitters** | **Unknown** | **Food & Beverage Workers** | **Cadres & Staff** | **Workers** | **Public Place Attendants** | **Seamen & Long-distance Drivers** | **Houseworkers & Unemployed** | **Teachers** | **Retirees** | **Migrant Workers** |
| --- | --- | --- | --- | --- | --- | --- | --- | --- | --- | --- | --- |
| **2023** | 9 | 2047 | 48 | 879 | 581 | 78 | 6 | 1179 | 180 | 1421 | 67 |
| **2024** | 13 | 3322 | 76 | 1443 | 474 | 49 | 6 | 2039 | 171 | 1999 | 113 |
| **2025** | 10 | 2471 | 76 | 1399 | 573 | 35 | 7 | 3865 | 266 | 2432 | 85 |
| **Total** | 32 | 7840 | 200 | 3721 | 1628 | 162 | 19 | 7083 | 617 | 5852 | 265 |
